# Drug prioritization using the semantic properties of a knowledge graph

**DOI:** 10.1038/s41598-019-42806-6

**Published:** 2019-04-18

**Authors:** Tareq B. Malas, Wytze J. Vlietstra, Roman Kudrin, Sergey Starikov, Mohammed Charrout, Marco Roos, Dorien J. M. Peters, Jan A. Kors, Rein Vos, Peter A. C. ‘t Hoen, Erik M. van Mulligen, Kristina M. Hettne

**Affiliations:** 1 Department of Human Genetics, Leiden University Medical Center, 2300 RC Leiden, The Netherlands; 2 Department of Medical Informatics, Erasmus MC, University Medical Center Rotterdam, 3000 CA Rotterdam, The Netherlands; 3 Faculty of Bioengineering and Bioinformatics, Moscow State University, 119234 Moscow, Russia; 4 Department of Methodology and Statistics, Maastricht University, 6200 MD Maastricht, The Netherlands; 5 Centre for Molecular and Biomolecular Informatics, Radboud Institute for Molecular Life Sciences, Radboud University Medical Center, 6500 HB Nijmegen, The Netherlands

**Keywords:** Data mining, Machine learning, Drug discovery

## Abstract

Compounds that are candidates for drug repurposing can be ranked by leveraging knowledge available in the biomedical literature and databases. This knowledge, spread across a variety of sources, can be integrated within a knowledge graph, which thereby comprehensively describes known relationships between biomedical concepts, such as drugs, diseases, genes, etc. Our work uses the semantic information between drug and disease concepts as features, which are extracted from an existing knowledge graph that integrates 200 different biological knowledge sources. RepoDB, a standard drug repurposing database which describes drug-disease combinations that were approved or that failed in clinical trials, is used to train a random forest classifier. The 10-times repeated 10-fold cross-validation performance of the classifier achieves a mean area under the receiver operating characteristic curve (AUC) of 92.2%. We apply the classifier to prioritize 21 preclinical drug repurposing candidates that have been suggested for Autosomal Dominant Polycystic Kidney Disease (ADPKD). Mozavaptan, a vasopressin V2 receptor antagonist is predicted to be the drug most likely to be approved after a clinical trial, and belongs to the same drug class as tolvaptan, the only treatment for ADPKD that is currently approved. We conclude that semantic properties of concepts in a knowledge graph can be exploited to prioritize drug repurposing candidates for testing in clinical trials.

## Introduction

Drug discovery is a time-consuming and costly process. Despite the exponential advancements in biological and information technologies, the number of new drugs introduced in the clinic has failed to advance similarly^1^. However, it is commonly known that some drugs can be used to treat multiple diseases. Identifying new indications for approved drugs, also known as drug repurposing, provides a relatively cheap and fast alternative to *de novo* drug discovery.

Smalheiser and Swanson were the first to demonstrate that the knowledge published in the biomedical literature could be computationally analyzed to identify and prioritize new drug therapies for diseases^2^. From the literature, Swanson had previously discovered that the pathological changes caused by Raynaud’s syndrome could be countered by the physiological changes caused by digesting fish oil, thereby suggesting a new treatment^3^. While Swanson’s original work was based on a relatively modest body of literature, the number of published research articles has continued to grow exponentially, making it even more costly and difficult for humans to obtain a comprehensive overview of the published knowledge that is relevant to their research^4,5^.

Beside the literature, biomedical databases provide another source of knowledge that can be analyzed. Their size and number have also shown an overwhelming growth. To improve reusability and interoperability between biomedical databases, their contents are increasingly being published as subject-predicate-object triples, which describe the relationships between the biological concepts represented in these databases. By semantically integrating the triples from different databases, biomedical knowledge can be connected across different sources in a knowledge graph. Knowledge graphs thereby enable computational analyses on a comprehensive representation of biomedical knowledge, supporting multiple stages of pharmacological research. While in early stages of pharmacological research new gene-disease associations may be suggested^6^, thereby facilitating the search for new targets, in a final stage the knowledge graph may be used to prioritize a list of drug candidate compounds.

Many knowledge-graph methods have already been developed to identify new drug therapies for diseases^7–14^. Most of these methods are based on similarity between drugs or diseases^7–10^. These methods count the number of concepts in the indirect paths, which are defined as sequences of two triples between two drugs, two diseases, or between a drug and a disease. The underlying assumption is that a high number of intermediate concepts indicates similarity between drugs, which are therefore likely to treat the same disease. For example, Yu *et al*. proposed a method that relied on overlapping networks of drug side effects and disease symptoms^14^. The networks were clustered to identify drug and disease modules, after which all drug-disease module pairs were connected. Based on known drug-disease combinations and local connectivity of modules, potential new drug therapies were predicted. A drawback of this method is that the suggested drug-disease combinations cannot distinguish between a drug-treatment relationship, a drug-side effect relationship, or a “does not treat” type of relationship.

Similarity-based methods often only include concepts from a limited number of semantic types, e.g. only proteins or side effects, thereby limiting their scope. Furthermore, Guney^15^ demonstrated that the performance of similarity-based methods is highly dependent on the availability of the drug in both the training set and the test set. If a drug is only represented in the test set, which could be the case for new or poorly characterized drugs, the performance of these methods drops drastically.

In other work, Guney *et al*.^11^ measured the distance in the graph between the target proteins of a drug, and disease proteins (i.e. the proteins coded for by the genes that are associated with a disease). The underlying assumption was that a short distance between drug targets and disease proteins meant that the drug was likely to treat the disease. Although this provided a coherent and plausible mechanism by which the efficacy of drugs could be explained, the study only used protein information, and the performance in determining the efficacy of drugs was moderate, with an area under the receiver operating characteristic curve (AUC) of 66%. Himmelstein *et al*.^12^ extracted paths of varying lengths between drugs and diseases from their knowledge graph. The predicates and semantic properties of the concepts in these paths were combined to create so-called metapaths (e.g. “Drug–*binds to*–Gene–*is associated with*–Disease”). Each metapath was represented as a binary feature, on which a machine-learning classifier was trained to classify whether a drug treats a disease. Alshahrani *et al*.^13^ transformed individual concepts and predicates in their graph to numeric vector representations with the RDF2vec tool, which were then used to train a classifier. However, due to the complexity of the transformation performed by the RDF2vec tool, it is impossible to reconstruct the relevant information that underlies a suggested relationship between a drug and a disease.

Here we leverage the semantic properties of concepts intermediate to drugs and diseases as features to classify and prioritize drug candidates. We utilize two kinds of semantic properties, both from the Unified Medical Language System (UMLS, https://www.nlm.nih.gov/research/umls/): semantic types, which are concept categories such as “Enzyme” or “Sign or Symptom”, and semantic groups, which are higher level abstractions of the semantic types (e.g. “Physiology”)^16^. The developed classifier is used to prioritize 21 candidate drugs for Autosomal Dominant Polycystic Kidney Disease (ADPKD), an inherited progressive kidney disease that leads to the growth of multiple cysts within the kidney and ultimately renal failure.

## Methods

### Knowledge Graph

We used the Euretos Knowledge Platform (EKP, https://www.euretos.com), a commercial knowledge graph that semantically integrates 200 biomedical knowledge sources. Three types of knowledge sources can be distinguished: (1) life-science databases, (2) textual and publication sources, and (3) semantic and ontological sources (http://www.euretos.com/files/EuretosSources2018.pdf). Please note that RepoDB is not one of these 200 sources. Mappings between the concepts in the different knowledge sources underlying the knowledge graph were made by matching their identifiers. All biomedical concepts in these knowledge sources, such as proteins, drugs, and diseases, are represented as vertices in a large graph. The edges represent relationships between the vertices, and specify the predicate and provenance information about the relationship. The knowledge graph represents each triple as two vertices, indicating the subject and the object, connected by an edge, indicating the predicate.

One of the knowledge sources within the EKP is the UMLS. The UMLS integrates the concepts and relationships from numerous biomedical terminologies, and assigns each concept to one or more of 137 semantic types. Each EKP concept has also been assigned to one of the 15 semantic groups defined by Bodenreider *et al*.^16^. For those concepts that were added to the EKP from other knowledge sources, semantic types and groups were manually assigned by Euretos based on the descriptions of their contents (e.g. the proteins in Uniprot were assigned the “Amino Acid, Peptide, or Protein” semantic type).

### RepoDB

We used RepoDB^17^ as a training and test set for our classifier. RepoDB consists of drug-disease combinations that have been approved or that have failed in clinical trials. The “Approved” drug-disease combinations in RepoDB (n = 6677) are obtained from DrugCentral, and are based on drug labels. RepoDB’s failed drug-disease combinations are based on unsuccessful clinical trials from the ClinicalTrials.gov database, which are subdivided in three categories: withdrawn (n = 648), indicating that the trial has been stopped before enrolling its first participant, suspended (n = 483), indicating that the trial has been stopped early but may be resumed at a later point, and terminated (n = 2754), indicating that the trial has been stopped and will not be resumed. To ensure that our negative subset only contained drug-disease combinations that have been tested in patients and that will not be re-examined in the foreseeable future, we decided to only use the “Terminated” subset and not the “Suspended” or “Withdrawn” subsets.

RepoDB represents drugs by DrugBank identifiers and diseases by UMLS concepts. A DrugBank identifier may have been mapped to multiple UMLS concepts (and therefore EKP concepts), e.g. for its active ingredient or its brand names. To ensure that we only extracted knowledge about the active ingredient of a drug from the EKP, the DrugBank identifiers from RepoDB were mapped to the UMLS concepts of their active ingredient.

Duplicate entries in RepoDB, including those caused by deprecated identifiers, were removed. In this study we focused on drug-disease combinations from RepoDB for which a direct path exists between the drug and the disease (e.g. drug A-*treats*-disease C), or for which the drug and the disease are indirectly connected via one intermediate concept (e.g. drug A-*interacts with*-protein B-*is associated with*-disease C). If the knowledge graph did not contain direct or indirect paths between a drug and a disease, the drug-disease combination was excluded from the set.

### Feature set and machine learning

**Figure 1:**
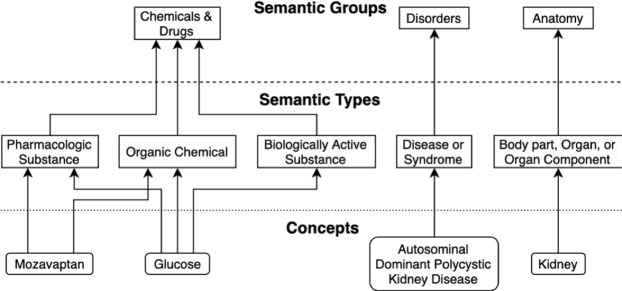
Example of correspondence between concepts, semantic types, and semantic groups. In total, the knowledge graph contains over 7 million concepts, each of which has been assigned one or more of 137 semantic types, and one of 15 semantic groups.

**Figure 2:**
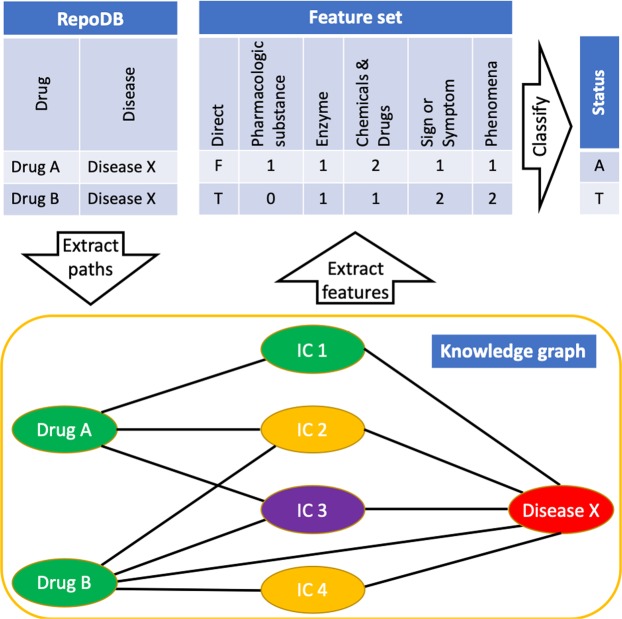
Overview of the feature creation process. RepoDB, which was used to train the classifier, contains drug-disease combinations whose status have been set to “Approved” or “Terminated” based on the results of clinical trials. Both the direct and the two-step indirect paths between the drugs and diseases are extracted from the knowledge graph. Based on the availability of a direct path between the drug and the disease, a binary feature is created. For the indirect paths, the frequencies of the semantic types and semantic groups of the intermediate concepts (IC) are used to create the features. In this figure, IC 1 has the semantic type pharmacologic preparation (i.e. a drug), IC 2 & 4 has Sign or Symptom, and IC 3 has Enzyme. Their semantic groups are Chemicals & Drugs for IC 1 and 3, and Phenomena for IC 2 and 4. Based on the extracted features, the classifier is trained/cross-validated to classify the status of each drug-disease combination as “Approved” (A) or “Terminated” (T).

For each drug-disease combination in our reference set the following features were extracted from the knowledge graph:

- A binary feature that indicates whether there was a triple which has the drug and the disease as subject and object (direct path);
- Numeric features that indicate the frequencies of the UMLS semantic types and semantic groups of the intermediate concepts (indirect paths);

For the frequency features, it is important to note that concepts can have multiple semantic types, but always belong to a single semantic group (Fig. 1). An overview of the feature creation and classification process using RepoDB is depicted in Fig. 2. All paths underlying the features were extracted via the EKP REST API.

We compared the classification performance of different machine learning algorithms on our feature set, based on multiple commonly used performance metrics. To provide insight into the variation of the classification performance we report the mean and standard deviation of the metrics based on 10 repeats of a 10-fold cross-validation experiment.

Because the majority of the diseases in RepoDB’s terminated combinations are cancers, and the majority of the diseases in the approved cases are not, we investigated whether our method was affected by this imbalance by training classifiers for cancers and non-cancers separately, for different class balances.

### Use case: Drug prioritization for ADPKD

ADPKD is an inherited progressive chronic kidney disease that leads to renal failure before the age of 60 in the majority of patients. It is a genetic disorder that causes renal tubules to become structurally abnormal, resulting in the development and growth of multiple cysts within the kidney. Currently, tolvaptan is the only approved drug treatment for this disease, and the tolvaptan-ADPKD combination is therefore included in RepoDB as a positive case. However, because tolvaptan has proven to be suitable for only a subset of ADPKD patients and has unfavorable side-effects, alternative drug treatments are searched for^18^. Furthermore, with clinical trials that test alternative drugs being scheduled or currently running, ADPKD is a suitable use case for candidate drug prioritization^19–24^.

Drug repurposing candidates for ADPKD that have been tested in preclinical studies were selected based on a manual review of the literature by two of the authors (T.B.M. and D.J.M.P, an ADPKD disease expert). Only review papers were included in the literature search^19–24^. These candidate drugs were subsequently prioritized using the classifier trained on RepoDB.

## Results

### RepoDB feature set

After removal of duplicate entries and drug-disease combinations for which no direct or indirect path was available, our set consisted of 8065 instances, out of the 9431 entries in RepoDB (86%). In total, there were 130 features based on the semantic properties of the intermediate concepts between the drugs and the diseases. Out of these 130, 13 were semantic groups, and 117 were semantic types.

One might hypothesize that the existence of a direct path between a drug and a disease would be predictive of an approved drug-disease combination. To test this hypothesis, we counted the number of direct paths in the knowledge graph for the terminated and approved subsets. In the approved subset 50% of the combinations had a direct path, while in the terminated subset 45% of the combinations had a direct path. These results indicate that the presence of a direct path has very limited discriminative value if used as a single feature. Table 1 shows the properties of the different subsets within our dataset.

**Table 1:** Number of unique drugs and diseases in the “Approved” and “Terminated” datasets. Each drug or disease could be part of multiple drug-disease combinations.

	Approved	Terminated	All
No. of Drugs	1407	373	1452
No. of Diseases	1111	719	1681
No. of non-cancers	787	226	933
No. of cancers	324	493	751
No. of drug-disease combinations	6044	2021	8065
No. of direct paths	3010	906	3916

### RepoDB cross-validation performance and feature importance

**Figure 3:**
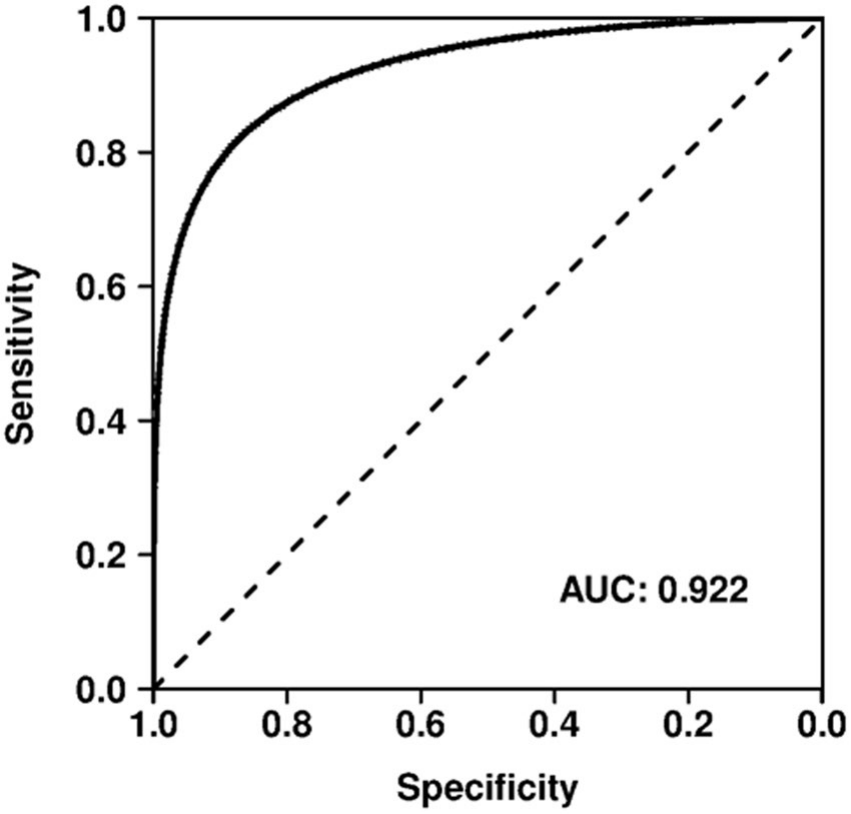
ROC curve of the 10-times repeated 10-fold cross-validation.

We created a feature set of the direct and indirect paths between the drugs and diseases present in the EKP using binary and numeric features for the approved and terminated subsets. Using this feature set, the classification performance of multiple machine learning algorithms was compared (Table 2). The random forest classifier achieved the best performance for all reported metrics, and was therefore chosen to perform further experiments with. Figure 3 shows the ROC curve for the random forest classifier, which achieved a mean AUC of 92.2% with a standard deviation of 1.2%. The repeated cross-validation experiments required 35 minutes at most, using 20 computing threads.

**Table 2:** Performance metrics achieved by each machine learning algorithm.

	Area under the ROC curve	Area under the precision-recall curve	F1	Accuracy	Kappa
Logistic Regression	81.6 (1.9)	90.8 (0.1)	87.6 (0.1)	79.8 (0.1)	35.4 (0.3)
Neural Network	89.1 (1.7)	95.0 (0.3)	90.9 (0.2)	86.0 (0.3)	60.7 (1.0)
SVM	88.4 (1.8)	94.8 (0.2)	90.6 (0.1)	84.7 (0.2)	51.1 (0.9)
CART	81.5 (2.0)	89.9 (0.0)	90.3 (0.1)	84.9 (0.1)	56.7 (0.3)
k-NN	88.2 (1.3)	94.2 (0.1)	90.7 (0.1)	85.5 (0.2)	57.9 (0.4)
Naïve Bayes	68.2 (1.8)	82.8 (0.1)	82.8 (0.1)	72.8 (0.1)	18.0 (0.2)
Random Forest	92.2 (1.3)	96.4 (0.1)	92.9 (0.0)	89.1 (0.1)	68.7 (0.2)

Values indicate mean and standard deviation (in %) of 10 repeats of a 10-fold cross-validation experiment.

**Figure 4:**
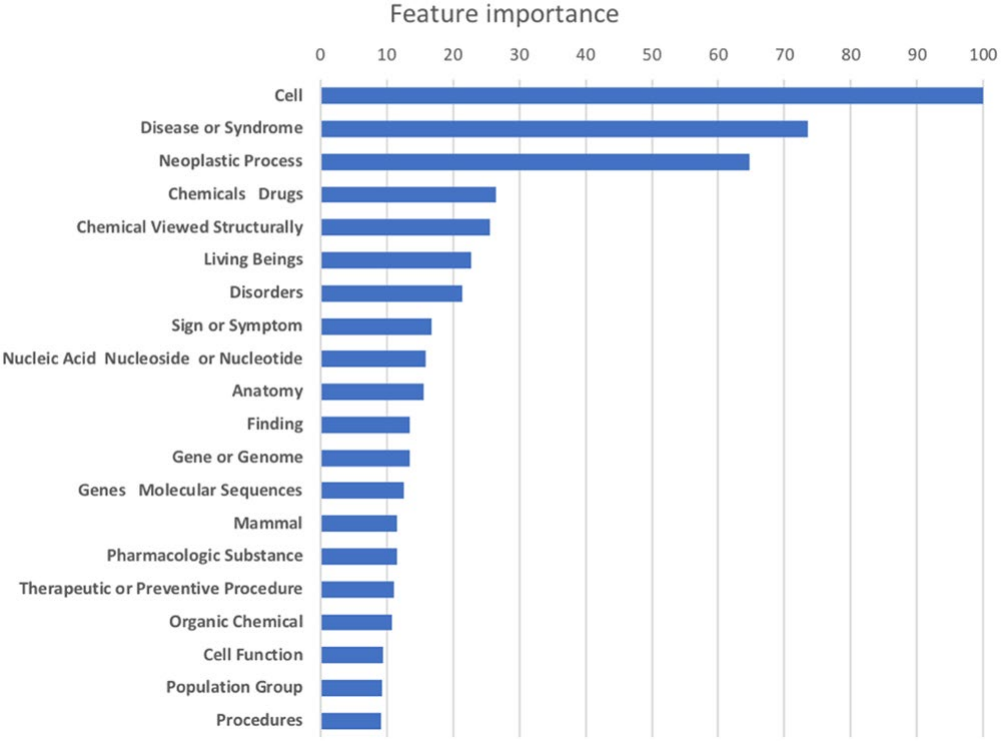
Individual feature importance scores, as calculated with the standard feature importance calculation function of the random forest algorithm. The scale of the scores have been normalized.

The importance of individual features was determined with the standard feature importance calculation function of the random forest algorithm. The semantic type “*Cell”* was the most important individual feature, followed by “*Disease or Syndrome*”, “*Neoplastic process*”, “*Chemicals & Drugs*”, and “*Chemical Viewed Structurally*”. Figure 4 lists the top-20 most important features. The binary feature for a direct path between a drug and a disease fell outside this top-20.

### Classification of cancer and non-cancer subsets

We attempted to determine the impact of the imbalance of cancer and non-cancer disease classes in the approved and terminated subsets on classification performance. Separate cross-validation experiments were performed for the cancer drug-disease combinations and for the non-cancer drug-disease combinations. The classifier trained on only non-cancers (AUC 84.2%) achieved a lower performance than the classifier trained on only cancers (AUC 91.6%). This performance difference may be caused by the different positive/negative ratios and sample sizes in the cancer and non-cancer subsets for the approved and terminated drug-disease combinations. When we subsampled the cancers (1519 approved combinations and 1590 terminated combinations) to get the same positive/negative ratio as the non-cancers (4525 approved combinations and 431 terminated combinations, for a 10:1 ratio), performance dropped to an AUC of 87.7%. The remaining 3.5 percent point difference may be caused by the fact that cancers on average are connected by more intermediate concepts than non-cancers: 662 and 466 respectively.

### Prioritization of drug candidates for ADPKD

We used the classifier that was trained on RepoDB to prioritize drugs which have been tested for ADPKD in preclinical studies. Based on a literature review, the experts identified 25 drug repurposing candidates for ADPKD. Table 3 shows a list of all candidates, along with a description of their mechanism of action and the review from which they were obtained. Three candidate drugs, teriflunomide, Genz-123346 and S31–201, were represented in the EKP, but were excluded because they did not have a direct or indirect path with ADPKD in the graph. Two candidate drugs, bosutinib and SKI-606, were included as separate results of the literature review, but because SKI-606 has bosutinib as its active ingredient, both drugs were represented by the concept for bosutinib during the prioritization of the drug candidates.

**Table 3:** Overview of drugs pre-clinically suggested for ADPKD.

Identifier	Name	Mechanism of action	Selected recent reference (PMID)
UMLS C0077274	Triptolide	intracellular calcium homeostasis	24560027
UMLS C2975283	Mozavaptan	Vasopressin V2 receptor antagonist	27578560
UMLS C2607958	Satavaptan	Vasopressin V2 receptor antagonist	18945944
UMLS C0028833	Octreotide	Somatostatin receptor agonist	26844873
UMLS C1872203	Pasireotide	Somatostatin receptor 2 agonist	24994926
UMLS C1174836	SKI-606	c-Src inhibitor	18385429
UMLS C1516119	Sorafenib	Raf kinase inhibitor	20810616
UMLS C0755562	U0126	MEK Inhibitor	18263604
UMLS C1831731	Bosutinib	Src/Bcr-Abl tyrosine kinase inhibitor	28838955
UMLS C0541315	Everolimus	FK506-binding protein 1 A inhibitor	25424440
UMLS C0072980	Sirolimus	FK506-binding protein 1 A inhibitor	29880342
UMLS C0025598	Metformin	Mitochondrial complex I (NADH dehydrogenase) inhibitor	21262823
UMLS C0071097	Pioglitazone	Peroxisome proliferator-activated receptor gamma agonist	28191533
UMLS C0289313	Rosiglitazone	Peroxisome proliferator-activated receptor gamma agonist	28191533
UMLS C0536217	Roscovitine	CDK inhibitor	23032260
UMLS C0025270	Menadione	Cdc25A	22155366
UMLS C0717758	Etanercept	TNF-alpha inhibitor	18552856
UMLS C0034283	Pyrimethamine	Stat3 inhibitor	21821671
ChemSpider 221421	S3I-201	Stat3 inhibitor	21821671
UMLS C1718383	Teriflunomide	Stat3 inhibitor	22155366
UMLS C1957685	Genz-123346	glucosylceramide synthase inhibitor	20562878
UMLS C0968934	HET-0016	20-HETE synthesis inhibitor	19129252
UMLS C0916207	TRAM-34	KCa3.1 inhibitor	18547995
UMLS C0010467	Curcumin	Multiple	21345977
UMLS C2935082	EX-527	SIRT1-specific inhibitor	23778143

The random forest classifier assigns a pseudoprobability (prediction score) between 0 and 100% to each candidate drug-ADPKD combination. Prediction scores close to 100% indicate a high confidence that a combination belongs to the approved class, whereas scores close to 0% indicate very low confidence. The cutoff between the approved and the terminated classes was set at 50%. The results are shown in Table 4.

**Table 4:** The prediction scores of our random forest classifier for the ADPKD drug repurposing candidates.

Name	Prediction score (%)	No. of intermediate concepts to ADPKD	No. of concepts the drug is connected to in the whole EKP
Mozavaptan	100.0	1	12
Satavaptan	93.0	2	17
HET-0016	92.6	4	67
Pasireotide	90.6	2	28
Bosutinib	88.6	9	1088
EX-527	73.8	6	75
Pioglitazone	68.2	53	1994
Octreotide	67.2	257	2133
Roscovitine	65.8	58	1027
Pyrimethamine	65.0	88	1062
TRAM-34	65.0	25	109
Etanercept	64.2	163	521
Triptolide	60.2	135	4128
Rosiglitazone	59.4	316	666
U0126	55.2	188	5891
Menadione	52.2	138	2822
Curcumin	46.0	359	2653
Metformin	42.4	343	3616
Everolimus	35.0	174	986
Sirolimus	27.0	376	5621
Sorafenib	19.0	205	1446

The classifier predicted that 17 out of the 21 drug candidates would be approved as treatment for ADPKD (prediction scores >50%). Mozavaptan achieved the highest prediction score (100%), followed by satavaptan (93.0%), HET-0016 (92.6%), and pasireotide (90.6%). Mozavaptan and satavaptan are vasopressin V2 receptor antagonists, as is the approved drug tolvaptan. Pasireotide is a somatostatin analogue. Another somatostatin analogue, octreotide, achieved a score of 67.2%. Mozavaptan, satavaptan, tolvaptan, pasireotide and octreotide all target cAMP signaling, which has a central role in ADPKD pathogenesis, with varying reported success^20^. The 20-HETE synthesis inhibitor HET-0016 has been suggested to mediate the proliferation of epithelial cells in the formation of renal cysts^25^.

The Raf kinase inhibitor sorafenib and two FK506-binding protein 1 A inhibitors, everolimus and sirolimus, had the lowest prediction scores (all below 40%). Sorafenib has been shown to block cAMP-dependent proliferation of human ADPKD cyst epithelial cells, but results in preclinical studies were ambiguous^26^. Sirolimus and everolimus were effective in preclinical studies, but had dose-limiting side effects in patients^27^.

#### Correlation of drug connectivity with prediction score

To investigate the role that the connectivity of a drug concept might have on its prioritization we calculated the correlations between (1) the prediction score and the number of drug-ADPKD intermediate concepts, and (2) the prediction score and the total number of concepts with which the drug concept was connected in the whole EKP (see Table 4). We found a strong significant negative correlation for the first comparison (Kendall’s rank correlation tau: −0.73, p < 0.001) and a moderate significant correlation for the second comparison (Kendall’s rank correlation tau: −0.52, p < 0.001). The negative correlations indicate that the classifier assigns lower prediction scores to drug-disease combinations with many intermediate concepts. The mean higher connectivity between cancers and drugs might contribute to this phenomenon.

#### Investigation of inclusion and exclusion of specific drugs in the training set

The tolvaptan-ADPKD combination is part of the “Approved” subset of RepoDB. To investigate the influence of this combination on the prioritization of the pre-clinically suggested candidate drugs, we excluded it from the set and trained a new classifier. With this classifier, tolvaptan achieved a prediction score of 79.2%, which was comparable with its prediction score on the full set (77.6%). These prediction scores of tolvaptan were lower than the classifier’s prediction scores of the other two vasopressin V2 receptors, mozavaptan (100%) and satavaptan (93.0%) (Table 4). Although this score is lower than for the other vasopressin V2 receptor antagonists, it shows that our method can confidently identify tolvaptan as an approved treatment for ADPKD. When tolvaptan was excluded from the training set, mozavaptan and satavaptan remained the top-two ranking drugs, with prediction scores of 100% and 95.0%, respectively. Based on these results, the high scores of mozavaptan and satavaptan cannot be explained by the clinical trial information available for the tolvaptan-ADPKD combination in RepoDB.

We performed a similar experiment for everolimus and sirolimus. Their poor prediction scores may be explained by the presence of sirolimus and everolimus in the “Terminated” RepoDB subset in combination with the generic form of ADPKD, “Polycystic Kidney Diseases”. We therefore excluded these combinations from the set and trained a new classifier. Everolimus and sirolimus then achieved prediction scores of 37.8% and 38.0% respectively, as compared with 35.0% and 27.0% on the full set, and retained their place in the ranking. The low scores of sirolimus and everolimus therefore cannot be explained by the clinical trial information in RepoDB about their combination with “Polycystic Kidney Diseases”.

### Qualitative analysis

**Figure 5:**
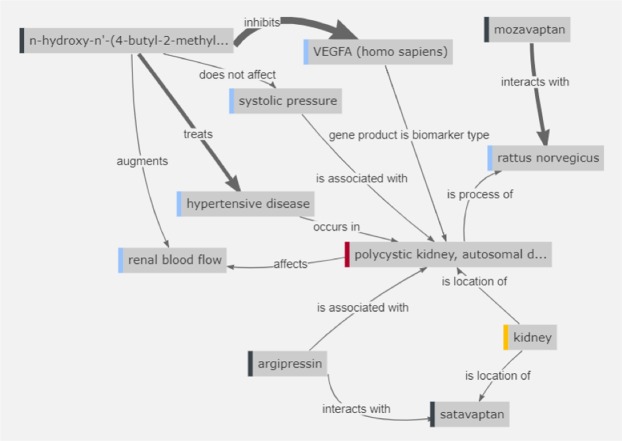
Simplified graph of the network of intermediate concepts between ADPKD and the top 3 drugs mozavaptan, satavaptan and HET-0016 (n-hydroxy-n’-(4-butyl-2-methyl phenyl)formamidine). The thickness of the arrows indicates the amount of underlying evidence (database entry or publication from the literature). For the sake of clarity, some paths were removed when creating the figure. The complete set of paths can be found at the github repository mentioned in the Data availability section.

The connection between mozavaptan and ADPKD in the EKP went through one intermediate concept: *Rattus norvegicus*. Two intermediate concepts, kidney and argipressin, connected ADPKD to satavaptan. Four intermediate concepts connected HET-0016 and ADPKD: VEGFA, hypertensive disease, renal blood flow, and systolic pressure, which suggests that HET-0016 may be used to treat the hypertensive component of ADPKD. Inhibition of 20-HETE production by HET-0016 has been shown to prevent and reverse adrenocorticotrophic hormone-induced hypertension but not dexamethasone-induced hypertension^28^, reduce cerebrovascular inflammation and oxidative stress, and improve vasomotor function in spontaneously hypertensive rats^29^. Figure 5 shows a simplified graph of the paths in the EKP between these drugs and ADPKD.

## Discussion

Our work demonstrates that the frequencies of semantic properties of intermediate concepts between drugs and diseases are powerful features to classify drug-disease combinations as “Approved” or “Terminated”. When evaluated with a repeated cross validation, our classifier achieved an excellent AUC of 92.2%. In our ADPKD use case we were able to rank 21 out of the 25 preclinical drug repurposing candidates that resulted from our literature review. When we prioritized these candidate drugs for ADPKD with our classifier, mozavaptan achieved the highest prediction score of 100%.

The comparison of drug prioritization methods is often impeded by the tendency of authors to only evaluate their methods on a locally available reference set. RepoDB was meant to remedy this, by being a publicly available gold-standard reference set against which all methods could be evaluated. To our knowledge, we are the first to use both its positive and negative cases to evaluate our method. Therefore, our results can only be indirectly compared to the work of Himmelstein *et al*.^12^. They used the approved drug-disease combinations from DrugCentral and ClinicalTrials.gov, the same databases underlying RepoDB, as external validation sets for their classifier, with which they obtained AUCs of 85.5% and 70.0%, respectively. Instead of the frequencies of the semantic types and groups of intermediate concepts, their binary features indicated the presence or absence of metapaths, which consisted of combinations of 16 unique predicates and 11 unique concept categories. These metapaths could vary in length, whereas we limited ourselves to indirect paths that contained a single intermediate concept. Both methods analyzed a wide range of biomedical knowledge, ranging from knowledge about diseases to cells to genes and proteins. Furthermore, both methods analyzed knowledge that was obtained from biomedical databases as well as the literature. However, whereas Himmelstein *et al*. integrated 29 knowledge sources with each other specifically for their drug prioritization task, we were able to save a considerable amount of time and effort by using an existing knowledge graph.

Two candidate drugs for ADPKD, bosutinib and SKI-606, were included as separate results of the literature review, even though they contain the same active ingredient. Because we only used concepts of active ingredients of drugs, we considered both of them to represent a single candidate. However, their separate inclusion in the literature review results may indicate that information about different variations of a drug is considered relevant by users. To accommodate such distinctions, our experiments could be repeated with the concept for a specific variation of a drug. Alternatively, all the concepts that represent the different variations of a drug could be grouped to repeat our experiments at a coarser granularity. Such groupings of concepts at different granularities are referred to as “scientific lenses”^30^. A different scientific lens could also be applied to ADKPD by grouping it with concepts for other variations of polycystic kidney disease. An obvious drawback of grouping concepts is that the details of specific variations of a drug or a disease are lost.

An important drawback of our machine learning method is the lack of straightforward comprehensibility of the classifier that is trained. Although the performance of the classifier is high, its use of the features may be counter-intuitive upon closer inspection. We encountered this phenomenon when we prioritized drug candidates for ADPKD. The top-ranked drug candidate, mozavaptan, was only supported by a minimal amount of information in the EKP, making it difficult to explain its prioritization. Nevertheless, mozavaptan was considered to be a viable candidate by the ADPKD expert. Overall, we found a significant negative correlation between the number of drug-ADPKD intermediate concepts and the prediction score. For example, for the somatostatin analogues octreotide and pasireotide, octreotide has a much lower prediction score and many more (n = 257) intermediate concepts to ADPKD than pasireotide (n = 2). This negative correlation might reflect the properties of the training set, where drugs on average have a higher number of intermediate concepts related to cancers than to non-cancers. Nonetheless, one could argue that the performance that is achieved during the cross-validation is high enough to rely on the classification results, however idiosyncratic their underlying assumptions may be.

In future research weights could be assigned to intermediate concepts based on network statistics. As was shown in the ADPKD use case, sometimes drugs and diseases are only connected by highly generic concepts. Assigning each intermediate concept a weight that is inversely proportional to its overall graph connectivity may diminish the contribution of generic concepts to the features.

Increasing the path length between the drugs and the diseases may help to substantiate a classification when drug-disease combinations are weakly connected. For example, there are seven intermediate concepts between mozavaptan and *Rattus norvegicus*, and 654 between ADPKD and *Rattus norvegicus*, which may offer further insight why mozavaptan was classified as the top candidate. Other potential benefits of including this information in the feature set may be an improved classification performance and an increased coverage of drug-disease combinations.

In summary, our method demonstrates that the variation and frequencies of semantic types and categories of intermediate concepts between drugs and diseases can be used as highly predictive features for classifying drug-disease combinations as “Approved” or “Terminated”. Because this task is a proxy for efficacy, our method is likely to be suitable for drug repurposing as well.

## Data Availability

All data and scripts can be found in the github repository (https://github.com/Wytz/Drug_repurposing).

## References

[1] Shim JS, Liu JO (2014). Recent advances in drug repositioning for the discovery of new anticancer drugs. International Journal of Biological Sciences.

[2] Smalheiser NR, Swanson DR (1998). Using ARROWSMITH: A computer-assisted approach to formulating and assessing scientific hypotheses. Comput. Methods Programs Biomed..

[3] Swanson DR (1986). Fish oil, Raynaud’s syndrome, and undiscovered public knowledge. Perspect. Biol. Med..

[4] Vardakas KZ, Tsopanakis G, Poulopoulou A, Falagas ME (2015). An analysis of factors contributing to PubMed’s growth. J. Informetr..

[5] Cook CE (2016). The European Bioinformatics Institute in 2016: Data growth and integration. Nucleic Acids Res..

[6] Hettne KM (2016). The implicitome: A resource for rationalizing gene-disease associations. PLoS One.

[7] Sirota M (2011). Discovery and preclinical validation of drug indications using compendia of public gene expression data. Sci. Transl. Med..

[8] Lee H (2012). Rational drug repositioning guided by an integrated pharmacological network of protein, disease and drug. BMC Syst. Biol..

[9] Daminelli S, Haupt VJ, Reimann M, Schroeder M (2012). Drug repositioning through incomplete bi-cliques in an integrated drug-target-disease network. Integr. Biol. (Camb)..

[10] Wang W, Yang S, Zhang X, Li J (2014). Drug repositioning by integrating target information through a heterogeneous network model. Bioinformatics.

[11] Guney E, Menche J, Vidal M, Barábasi AL (2016). Network-based in silico drug efficacy screening. Nat. Commun..

[12] Himmelstein DS (2017). Systematic integration of biomedical knowledge prioritizes drugs for repurposing. Elife.

[13] Alshahrani Mona, Khan Mohammad Asif, Maddouri Omar, Kinjo Akira R, Queralt-Rosinach Núria, Hoehndorf Robert (2017). Neuro-symbolic representation learning on biological knowledge graphs. Bioinformatics.

[14] Yu L, Ma X, Zhang L, Zhang J, Gao L (2016). Prediction of new drug indications based on clinical data and network modularity. Sci. Rep..

[15] Guney E (2016). Reproducible drug repurposing: When similarity does not suffice. Pacific Symp. Biocomput..

[16] McCray AT, Burgun A, Bodenreider O (2001). Aggregating UMLS semantic types for reducing conceptual complexity. Stud. Health Technol. Inform..

[17] Brown AS, Patel CJ (2017). A standard database for drug repositioning. Sci. Data.

[18] Torres VE (2012). Tolvaptan in patients with autosomal dominant polycystic kidney disease. N. Engl. J. Med..

[19] Torres VE, Harris PC (2007). Polycystic kidney disease: genes, proteins, animal models, disease mechanisms and therapeutic opportunities. J. Intern. Med..

[20] Irazabal MV, Torres VE (2013). Experimental therapies and ongoing clinical trials to slow down progression of ADPKD. Curr. Hypertens. Rev..

[21] Chang, M.-Y. & Ong, A. C. M. Mechanism-based therapeutics for autosomal dominant polycystic kidney disease: recent progress and future prospects. *Nephron. Clin. Pract*. **120**, c25–34; discussion c35 (2012). 10.1159/000334166 22205396

[22] Belibi FA, Edelstein CL (2010). Novel targets for the treatment of autosomal dominant polycystic kidney disease. Expert Opin. Investig. Drugs.

[23] Pan J, Seeger-Nukpezah T, Golemis EA (2013). The role of the cilium in normal and abnormal cell cycles: emphasis on renal cystic pathologies. Cell. Mol. Life Sci..

[24] Stayner, C., Brooke, D. G., Bates, M. & Eccles, M. R. Targeted Therapies for Autosomal Dominant Polycystic Kidney Disease. *Curr. Med. Chem*. **25** (2018). 10.2174/0929867325666180508095654 29737248

[25] Park F, Sweeney WE, Jia G, Roman RJ, Avner ED (2008). 20-HETE mediates proliferation of renal epithelial cells in polycystic kidney disease. J. Am. Soc. Nephrol..

[26] Yamaguchi T, Reif GA, Calvet JP, Wallace DP (2010). Sorafenib inhibits cAMP-dependent ERK activation, cell proliferation, and *in vitro* cyst growth of human ADPKD cyst epithelial cells. Am. J. Physiol. Renal Physiol..

[27] Ruggenenti P (2016). Effect of Sirolimus on Disease Progression in Patients with Autosomal Dominant Polycystic Kidney Disease and CKD Stages 3b-4. Clin. J. Am. Soc. Nephrol..

[28] Zhang Y (2009). The role of 20-hydroxyeicosatetraenoic acid in adrenocorticotrophic hormone and dexamethasone-induced hypertension. J. Hypertens..

[29] Toth P (2013). Treatment with the cytochrome P450 ω-hydroxylase inhibitor HET0016 attenuates cerebrovascular inflammation, oxidative stress and improves vasomotor function in spontaneously hypertensive rats. Br. J. Pharmacol..

[30] Batchelor C (2014). Scientific Lenses to Support Multiple Views over Linked Chemistry Data. Semant. Web – ISWC 2014.

